# Bioinformatics analysis of *CYP1B1* mutation hotspots in Chinese primary congenital glaucoma patients

**DOI:** 10.1042/BSR20180056

**Published:** 2018-07-06

**Authors:** Zhiying Ou, Guangjian Liu, Wenping Liu, Yehua Deng, Ling Zheng, Shu Zhang, Guangqiang Feng

**Affiliations:** 1 Institute of Pediatrics, Guangzhou Women and Children’s Medical Center, Guangzhou Medical University, Guangzhou, Guangdong, China; 2 Department of Medical Devices, Guangdong Food and Drug Vocational College, Guangzhou, Guangdong, China; 3 Department of Ophthalmology, Guangzhou Women and Children’s Medical Center, Guangzhou Medical University, Guangzhou, Guangdong, China; 4 Department of Ophthalmology, Jieyang People’s Hospital, Jieyang Affiliated Hospital, Sun Yat-sen University, Jieyang, Guangdong, China

**Keywords:** CYP1B1, computational biochemistry, molecular dynamics, Mutation, Primary congenital glaucoma, protein structure

## Abstract

Primary congenital glaucoma (PCG) is an inherited blinding eye disease. The *CYP1B1* gene was identified as a causal gene for PCG, and many mutations have been found, but no studies have focussed on the molecular epidemiology of *CYP1B1* in Chinese populations. We aimed to explore the *CYP1B1* mutation hotspots in Chinese PCG patients and the possible impact of these mutations on the protein structure and function. First, we performed a meta-analysis on seven datasets of Chinese populations and found L107V and R390H to be the most common *CYP1B1* mutations with allele frequencies of 3.19% and 3.09%, respectively. Then, a series of bioinformatics tools were applied to determine the sequence conservative properties, model the 3D structures, and study the dynamics changes. L107 and R390 are highly conserved residues in close proximity to the hemoglobin-binding region and the active site cavity (ASC), respectively. The mutations changed the distribution of hydrogen bonds and the local electrostatic potential. Long-term molecular dynamics (MD) simulations demonstrated the destabilization of the mutant proteins, especially at the ASC, whose solvent-accessible surface areas (SASAs) were significantly decreased. Compared with the wild-type (WT) protein, the overall structures of the mutants are associated with subtle but significant changes, and the ASC seems to adopt such structures that are not able to perform the WT-like functionality. Therefore, L107V and R390H might be the most important pathogenic mutations in Chinese PCG patients.

## Introduction

Primary congenital glaucoma (PCG) is an eye disease that affects children between the ages of birth and 3 years [1]. Untreated PCG is a major cause of childhood blindness. PCG is thought to arise due to dysplasia of the eye anterior chamber angle and trabecular meshwork during embryo development. Approximately 50% of patients show symptoms at birth, and 80% of patients are confirmed at 1 year. Amongst these, 65% of patients are male and 70% of patients have symptoms in both the eyes [2].

Population and regional disparities in the incidence of congenital glaucoma are apparent, with an incidence rate of 1:10000 (1:5000–1:22000) in Western countries, 1:3300 in the Indian Andhra Pradesh area, and 1:2500 in Middle Eastern countries [3–5]. The Gypsy population in Slovakia has the highest incidence of 1:1250, which might be due to the higher incidence of consanguinity. This point of view is further supported by the fact that the consanguinity rate in the parents of PCG patients is significantly higher than those of secondary congenital glaucoma patients [6].

Family studies indicate that the most PCG patients fit the autosomal recessive pattern. The genetic heterogeneity of this disorder implies that PCG is caused by multiple genes. However, mutations in *CYP1B1* have been considered the most frequent cause, with a penetrance of 40–100% [7]. Since *CYP1B1* was first identified as a causal gene for PCG [6], more than 80 mutations in this gene have been detected in PCG patients around the world, including more than 20 mutations in Chinese PCG patients [8,9]. The enzyme encoded by *CYP1B1* plays an important role in the development of the anterior segment and the regulation of corneal transparency and aqueous humor secretion [6].

Given the population heterogeneity of PCG and the increasing number of mutations found in *CYP1B1*, it is of great importance to summarize and analyze common *CYP1B1* mutations for disease prevention and diagnosis. As far as we know, no studies have focussed on the meta-analysis of *CYP1B1* mutations in Chinese PCG patients or on the possible impact of these mutations on protein structure and function. Therefore, in the present study, we first explored the mutational hotspots of *CYP1B1* in Chinese populations, and inferred the relationships between the mutations and protein function by analyzing the evolutionarily conserved sequence, modeling tertiary structures, and conducting long-term molecular dynamics (MD) simulations.

## Materials and methods

### Literature inclusion and exclusion criteria

Potential literature citations were retrieved from PubMed and three Chinese medical databases (CNKI, Wanfang, and VIP), by searching the keywords ‘Chinese’, *‘CYP1B1’* and ‘primary congenital glaucoma’. We selected literature based on the following inclusion and exclusion criteria. The inclusion criterion for PCG and the *CYP1B1* gene variation in our study included diagnosis of patients with PCG within 3 years of age, sporadic origins of all patients, a number of the total cases that is no less than 13 for PCG *CYP1B1* gene mutation screening, and reports on screening methods, mutation sites, and mutation frequency. The exclusion criteria was as follows: (i) PCG that merged with other eye diseases, other ocular or systemic diseases caused by the increased intraocular pressure of glaucoma, and (ii) similar studies published in different periods of time from the same research group and the latest research results that have been used before.

Data that met the selection criteria were extracted independently by two investigators, and the sample size, mutation sites, amino acid changes in the circumstances, and mutation detection rate variability were included. Differences in data extraction were discussed and agreed on by both the investigators.

### Meta-analysis for *CYP1B1* gene variants

All literature on PCG *CYP1B1* gene mutations in Chinese PCG patients published before December 2016 was surveyed. Meta-analysis was used to merge data from all research results. The frequencies of the *CYP1B1* gene mutation were statistically analyzed. The top two mutations with high frequency were selected for further protein sequence, structure, and function analysis.

### Conservative analysis for CYP1B1 protein residue variation

The normal human CYP1B1 sequence in the NCBI protein database (NP_000095.2) was extracted. Twenty sequences were aligned for conservation analysis; sequence logo graphics were generated using SeqLogo (ver. 2.13) as introduced by Schneider and Stephens [10]. The 20 sequences are as follows: CYP1B1, CYP1A1, CYP1A2, CYP2B6 and CYP2C9 from *Homo sapiens*; CYP1B1 from *Pan troglodytes, Papio cynocephalus, Macaca mulatta, Bos taurus, Canis lupus familiaris, Mus musculus, Rattus norvegicus, Danio rerio, Ictalurus punctatus, Oreochromis niloticus, Gallus, Oryzias melastigma, Phoca sibirica*, and *Fundulus heteroclitus*; and CYP1A1 from *Lagenorhynchus acutus*.

### Tertiary structure analysis

The crystal structure of wild-type (WT) CYP1B1 was downloaded from RCSB (http://www.rcsb.org/) with the PDB code 3PM0 [11]. The 3D structures of mutant CYP1B1 were modeled per homology by replacing the corresponding side chain in WT CYP1B1 and subsequently performing 2000 steps of minimization, while all the atoms except the mutated residue were fixed and another 3000 steps with all the atoms free. Finally, the protein 3D structures were validated, and the hydrogen bonds were analyzed with VMD [12]. A hydrogen bond was defined if the donor–acceptor distance was <3.5 Å, and the donor–hydrogen acceptor angle was >150°. The secondary structure information was analyzed using the PDBsum server (http://www.ebi.ac.uk/pdbsum/) [13]. The electrostatic properties of WT and R390H mutants were investigated with APBS [14] and displayed using VMD. The pqr input file required to run APBS was prepared using PDB2PQR 2.0.0 [15]. The electrostatic potential was calculated by solving the linearized Poisson–Boltzmann equation at 310 K, using dielectric constant values of 2 and 78.54 for protein (solute) and solvent, respectively.

### MD simulations

The WT and mutant CYP1B1 monomer co-ordinates obtained in the previous section were used as starting structures for initiating the MD simulations. The NAMD 2.6 [16] program along with the CHARMM36 all-atom force field [17,18] were used for MD simulations. First, each of the three structures was solvated with TIP3P water molecules in a rectangular box (roughly 6.4 × 7.2 × 7.5 nm) and neutralized at a 150 mM salt concentration, respectively, to mimic the real physiological environment [19]. Then, each system was energy-minimized for 2000 steps with all the protein atoms constrained and for another 5000 steps with all the atoms free. After that, each system was heated up to 310 K in 100 ps and equilibrated for 100 ns. The temperature was held at 310 K using Langevin dynamics, and the pressure was held at 1 atmosphere by the Langevin piston method. A 12-Å cutoff for electrostatic and van der Waals interactions was adopted, along with cMAP correction for backbone and particle mesh Ewald algorithm for electrostatic interaction [20].

All analyses were performed with VMD tools [12]. Time courses of the Cα RMSD illustrated the conformational stabilities of the structures. The Cα root mean square fluctuation (RMSF) patterns marked the local structural flexibilities. The solvent-accessible surface area (SASA) with a 1.4-Å probe radius was calculated to measure the degree of exposure of the active sites.

## Results

### Meta-analysis of CYP1B1 mutations in Chinese PCG patients

Seven published studies [21–27] satisfied the inclusion and exclusion criteria, and 352 PCG cases with complete clinical data were included in the meta-analysis (Table 1). Mutations L107V and R390H of CYP1B1 were ranked the top two by the mutation frequency. The L107V mutation was first reported in a Chinese PCG population [22] and had the highest frequency of 3.19% amongst all the PCG with CYP1B1 mutations. The R390H mutation is a common mutation reported frequently in the literature, and its mutation frequency was 3.09% in Chinese patients. The frequencies of other loci ranged from 0.82 to 2.44%.

**Table 1:** CYP1B1 gene mutation frequency in Chinese PCG patients

Mutation type	Nucleotide change	Amino acid	Positive cases/Total cases	First reported in China	Positive cases	Total cases	Mutation frequency (%)
Missense mutation	g.4124C>G	L107V	2/116 [22]; 5/122 [26]; 1/13 [21]	No	8	251	3.19
	g.8006G>A	R390H	2/116 [22]; 4/41 [24]; 1/16 [25]; 1/44 [27]; 2/122 [26]	No	10	324	3.09
	c.1007C>A	S336Y	1/41 [24]	Yes	1	41	2.44
	c.1412T>G	I471S	1/41 [24]	Yes	1	41	2.44
	g.7924G>T	V363F	1/44 [27]	Yes	1	44	2.27
	g.7946C>T	P370L	1/44 [27]	Yes	1	44	2.27
	g.8254G>A	E473K	1/44 [27]	Yes	1	44	2.27
	g.4642C>G	H279Q	1/44 [27]	Yes	1	44	2.27
	g.4677A>G	D291G	3/112 [22]; 2/122 [26]	Yes	5	234	2.14
	g.4763G>T	V320L	1/44 [27]; 2/122 [26]	No	3	166	1.81
	g.8242C>T	R469W	1/41 [24]; 1/16 [25]; 1/122 [26]	No	3	179	1.68
	g.4338T>A	V178E	2/122 [26]	Yes	2	122	1.64
	g.4493G>A	E230K	2/122 [26]	Yes	2	122	1.64
	g.4449G>T	S215I	1/116 [22]; 2/122 [26]	No	3	238	1.26
	g.3985C>G	Z60M	1/116 [22]	Yes	1	116	0.86
	g.4089T>C	V95A	1/116 [22]	Yes	1	116	0.86
	g.4157C>T	P118S	1/116 [22]	Yes	1	116	0.86
	g.4206T>C	F134S	1/116 [22]	Yes	1	116	0.86
	g.4413A>G	N203S	1/116 [22]	Yes	1	116	0.86
	g.4664G>A	A287S	1/116 [22]	Yes	1	116	0.86
	g.4761A>G	N319S	1/116 [22] [22]	Yes	1	116	0.86
	g.7925T>A	V363D	1/116 [22]	Yes	1	116	0.86
	g.4397G>A	V198I	1/116 [22]	No	1	116	0.86
	g.8147C>T	P437L	1/116 [22]	No	1	116	0.86
	g.7927G>A	V364M	1/116 [22]; 1/122 [26]	No	2	238	0.84
	g.8168G>A	R444Q	1/116 [22]; 1/122 [26]	No	2	238	0.84
	g.7940G>T	R368L	1/116 [22]; 1/122 [26]	Yes	2	238	0.84
	g.3836T>C	W11R	1/122 [26]	Yes	1	122	0.82
	g.4151G>T	D116Y	1/122 [26]	Yes	1	122	0.82
	g.4322G>A	E173K	1/122 [26]	Yes	1	122	0.82
	g.4509T>C	V235A	1/122 [26]	Yes	1	122	0.82
	g.8137T>C	W434R	1/122 [26]	Yes	1	122	0.82
	g.8167C>T	R444stop	1/122 [26]	Yes	1	122	0.82
Delete mutation	g.3972delC	A56GGHX→59 stop	1/116 [22]	Yes	1	116	0.86
	c.828delC	F276Ffsx1	1/41 [24]	Yes	1	41	2.44
	c.726-747del22bp	D242Dfsx28	1/41 [24]	Yes	1	41	2.44
	g.4022delTC	73ORF→221 stop	1/122 [26]	Yes	1	122	0.82
Insert mutation	g.4168_4169 ins (GACCGGCCGGCCTTCGCC)	A121_S122 ins (DRP AFA)	1/116 [22]	Yes	1	116	0.86
Delete and Insert	g.8209_8213del (AGCAG) ins (TTGTTGAAAAA)	S458_R459del, ins LLKK	1/116 [22]	Yes	1	116	0.86

### Conservation analysis for CYP1B1 mutations

**Figure 1:**
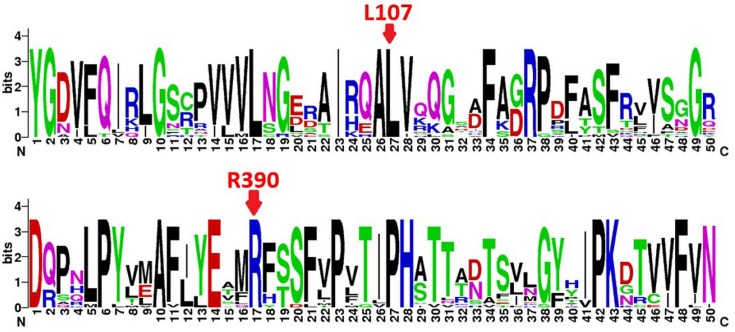
Conservation analysis for CYP1B1 protein residue sequence The two red arrow keys indicate the two highest Chinese mutation frequency loci L107 and R390 calculated in Table 1. They are both conserved residues in the P450 superfamily. The protein sequences used for this conservative analysis are CYP1B1*-Homo sapiens* NP_000095.2; CYP1B1*-Pan troglodytes* JAA44559.1; CYP1B1*-Papio cynocephalus* ACR60427.1; CYP1B1*-Macaca mulatta* NP_001253797.1; CYP1B1*-Bos taurus* NP_001179223.1; CYP1B1*-Canis lupus familiaris* NP_001153156.1; CYP1B1*-Mus musculus* NP_034124.1; CYP1B1*-Rattus norvegicus* NP_037072.1; CYP1B1*-Danio rerio* AAW52507.1; CYP1B1*-Ictalurus punctatus* NP_001187243.1; CYP1B1*-Oreochromis niloticus* NP_001266505.1; CYP1B1*-Gallus gallus* XP_004941356.1; CYP1B1*-Oryzias melastigma* AGN04320.1; CYP1B1*-Phoca sibirica* BAF58169.1; CYP1B1*-Fundulus heteroclitus* ACO51071.1; CYP1A1*-Lagenorhynchus acutus* AAV34440.1; CYP1A2*-H. sapiens* NP_000752.2; CYP2B6*-H. sapiens* NP_000758.1; CYP2C9*-H. sapiens* NP_000932.3; and CYP1A1*-H. sapiens* NP_000490.1.

We evaluated the pathogenicity of the mutations by aligning 20 protein sequences of cytochrome P450 superfamily, such as human CYP1B1, CYP1A1, CYP1A2, CYP2B6 and CYP2C9, as well as CYP1B1 from other species. Sequence conservation of amino acid residues was quantitatively presented in bits of information by using sequence logo graphics. The results indicated that both L107 and R390 were conserved in the evolution of the P450 protein family amongst different species (Figure 1).

### Changes in the static structures

Homology modeling was performed from the crystal structure of WT CYP1B1 to elucidate the possible structural effects of the mutations L107V and R390H. Ramachandran plots of main chain torsion angles indicated that most residues have a reasonable conformation (Supplementary Figure S1), demonstrating that the modeled mutant structures are of good quality. The all-atom RMSD with respect to WT CYP1B1 was 1.25 Å for L107V and 1.62 Å for R390H.

**Figure 2:**
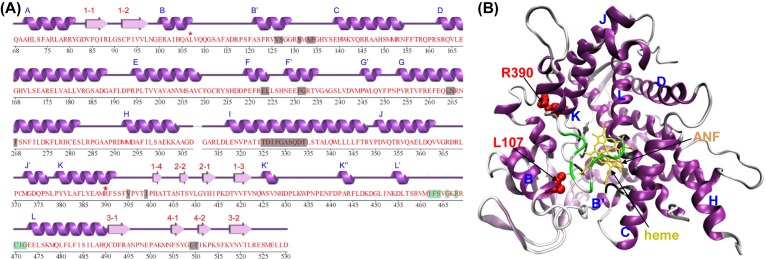
The secondary and tertiary structures of human CYP1B1 protein (**A**) The secondary structure diagram determined by the define secondary structure of proteins (DSSP) algorithm was obtained from the PDBsum website. Helical and extended secondary structures are shown in the purple and pink type, respectively, and are labeled alphabetically and numerically above the sequence. Gray highlighted regions are the active site amino acid residues. Purple highlighted regions are the ferrous hemoglobin-binding sites. The mutation sites are marked with red asterisks. (**B**) The tertiary structure was colored according to (A). The mutation sites are shown in the Van der Waals (VDW) spheres. The inhibitor α-naphthoflavone (ANF) and heme are represented as licorices.

As shown in the secondary structure diagram (Figure 2A), the amino acid residues residing in the active site cavity (ASC) of CYP1B1 mainly lie in α-helices and another functionally important region, the heme binding region (HBR), lies in an interhelical loop. The mutation sites L107 and R390 are located in the B- and K-helix, respectively. In the tertiary structure, L107 and R390 are close to both the ASC and HBR (Figure 2B). Therefore, these two important functional regions might be affected by the mutations L107V and R390H.

**Figure 3:**
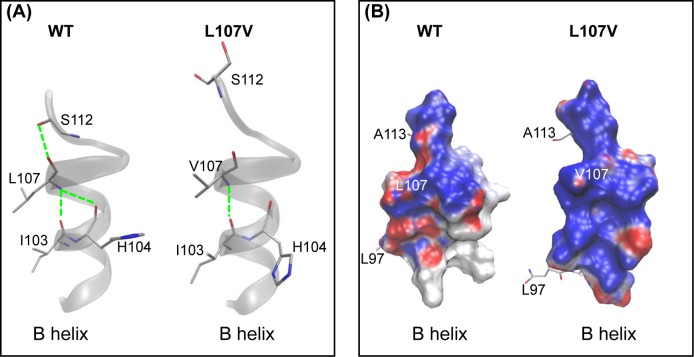
Molecular model of the B-helix showing the 107th residue at the helix end (**A**) The network of intrahelical hydrogen bond interactions involved by the 107th residue is shown in the WT CYP1B1. The L107V mutation causes a significant loss of these interactions, resulting in protein misfolding. (**B**) The local electrostatic potentials of the B-helix in WT and L107V. Potentials less than −10 kT/e are colored in red, and those greater than +10 kT/e are depicted in blue. The positive charges of the B-helix in L107V are significantly higher than those in the WT.

Molecular modeling analysis of the L107V mutant showed that the mutation might change the stability of the B-helix. In WT CYP1B1, a network of three intrahelical hydrogen bonds exists between L107 and the other three residues I103, H104, and S112. The presence of valine at position 107 in the L107V mutant causes a change in the conformation of the protein backbone, resulting in a significant loss of two hydrogen bonds with H104 and S112 (Figure 3A), which may have disturbed the end of the B-helix structure. This result obtained from the residue interaction network is supported by the electrostatic potential analysis from APBS, which shows a change from electronegativity to electropositivity (Figure 3B). Although L107V can be considered a conservative mutation with respect to the functionality (leucine and valine are both hydrophobic), structurally, it is nonconserved because valine is a β-branched amino acid. A β-branched amino acid can restrict the conformational space of the protein backbone, causing bulkiness in close proximity to the main chain [28], as observed in the case of the L107V mutant.

**Figure 4:**
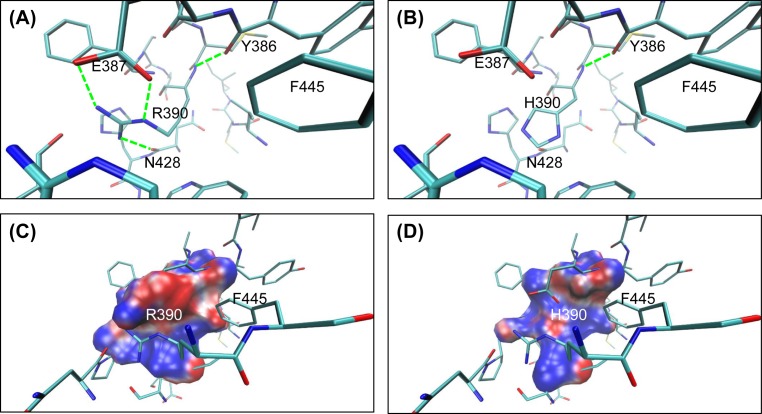
The hydrogen bonds and the local electrostatic potential before and after the 390th residue variation in human CYP1B1 (**A**,**B**) The hydrogen bonds attributed for the WT and the mutant. R390 involves four hydrogen bonds, while only one hydrogen bond is formed between H390 and the surrounding residues. The hydrogen atoms were removed for clarity. (**C**,**D**) The local electrostatic potentials within 3 Å of the 390th residue for the WT and the mutant. Colors are shown the same as those in Figure 3. The positive charges around H390 are significantly higher than those of R390.

With respect to the mutation R390H, although both arginine and histidine are hydrophilic and alkaline amino acids, they are significantly different in the formation of hydrogen bonds. Four hydrogen bonds around R390 are formed between R390 and Y386, E387, and N428. However, only one hydrogen bond forms between H390 and Y386 (Figure 4A,B). Furthermore, the electrostatic potential analysis from APBS showed that the amino acid substitution R390H radically changes the electrostatic potentials around the mutated position from significantly negative to slightly positive (Figure 4 C,D). This change from electronegativity to electropositivity supports our results obtained from the hydrogen bond network and would probably change the residue interaction in the ASC.

### MD simulation studies

To investigate how the mutation propagates into the protein structure leading to a loss of function, rather than only some qualitative aspects of the possible deleterious effects as described above, the state-of-the-art MD simulations were applied to each of the WT and mutant proteins for 100 ns. The snapshots were saved at every successive 2 ps from the start of the simulation, totaling 50000 for each protein, which were used to analyze the time evolutions of various structural properties on the whole protein as well as the ASC.

**Figure 5:**
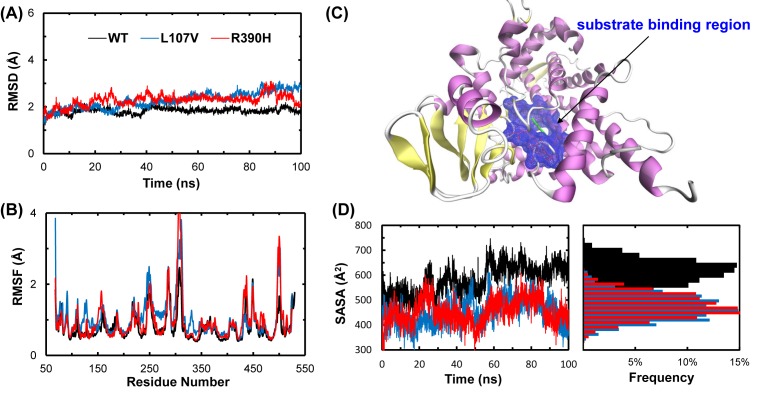
Stabilities of WT and mutant CYP1B1 during MD simulations (**A**) RMSD of heavy atoms for the WT, L107V, and R390H CYP1B1. (**B**) RMSF of each residue for the WT, L107V, and R390H CYP1B1. (**C**) The SASA (blue mesh) of the amino acid residues residing in the ASC indicated in Figure 2. Green represents ANF.(**D**) Variation and distribution of SASA of the ASC for the WT, L107V, and R390H CYP1B1.

The overall structural deviation of the WT and mutant proteins from their starting structures is indicated by time courses of the Cα RMSD (Figure 5A). Although the starting structures of the WT and mutants were almost identical, the trajectories shown in Figure 5A do not take precisely the same course. We observed that all the three structures stabilized after 20 ns, but the levels of RMSDs of the mutants increased remarkably by the mutation L107V or R390H, suggesting a mutation-induced destabilization. The distribution of Cα RMSF values calculated with the data collected after RMSD stabilization is shown in Figure 5B. RMSF distribution showed that L107V and R390H were more flexible than the WT in some of the residues and might induce some local conformational changes. This is in accordance with the results obtained from RMSD.

Next, we calculated the SASA of the amino acid residues residing in ASC to study the effect of mutations on this functionally important region (Figure 5C). Both the mutants showed a reduction in the SASA of their ASCs (Figure 5D). The L107V and R390H mutations appear to be a disturber of substrate binding, altering the distribution of the SASA of ASC in WT CYP1B1. For WT CYP1B1, the SASA of ASC peaks was approximately 640 Å^2^, while those of L107V and R390H peaked at approximately 460 and 440 Å^2^, as shown in the probability distribution of the SASA (Figure 5D). These results indicate that L107V and R390H are characterized by a distorted substrate-binding pocket, smaller than that found in the WT.

## Discussion

To date, genetic linkage studies have indicated that PCG is a heterogeneous disease that is mainly mapped on to the locus *GLC3A* and the *CYP1B1* gene, which harbors more than 80 mutations in PCG amongst different racial groups [29,30]. Since the relationship was apparent between the *CYP1B1* gene variants and the eye angle tissue histology or the clinical phenotype, PCG treatment programs could be established according to the mutation screening. In the present study, we summarized the molecular epidemiological data and found two common *CYP1B1* gene mutations in Chinese populations. Then, we linked these mutations to protein function with a series of bioinformatics tools.

The detection rate of the *CYP1B1* gene mutations was 15.9% (56/352) in our study (Table 1), which is close to the former reports (17%) in Chinese PCG patients [21,22,31]. The mutation frequencies of L107V and R390H were highest amongst all the known variable sites according to our meta-analysis, indicating that these two mutations were closely associated with PCG pathogenesis. In addition, L107V was also found in Korean PCG patients with a frequency of 2.4% (4/170) [32]. Therefore, we concluded that L107V might be an East Asian PCG pathological mutation.

Generally, key residue substitutions occurred in catalytic/substrate-binding sites would cause a loss of protein function. However, both L107V and R390H are neither in the ASC nor in the HBR of CYP1B1, although they are indeed close to these regions (Figure 2). It is argued that such mutations might confer deleterious effects through changes in some structural characteristics such as the disruption of intramolecular hydrogen bond networks, the change in electrostatic potential distribution etc. In general, any amino acid substitution can affect the protein structure to some extent, depending on the nature of the amino acid being replaced and newly introduced, as well as the mutation site. Regarding the pathogenic mutations investigated in the present study, we found that they not only transformed local structures but were also associated with some structural changes in the functionally important region.

The P450 superfamily, including CYP1B1, is characterized by many highly conserved core structures comprising approximately 14 α-helices and 4–6 β-sheets. From the results presented in the current paper, we can speculate the role played by the conserved L107 in the B-helix. In WT CYP1B1, L107 is involved in a compact network of intrahelical hydrogen bonds (Figure 3). Upon mutation into valine, two-thirds of hydrogen bonds are broken; thus, the continuity of the B-helix might be destroyed.

E387, R390, and R444 of CYP1B1 form a special structure known as the glutamic acid-arginine-arginine triad, which is a conserved folding core in the CYP1 family [33–35]. In addition, E387**R390 has also been recognized as an absolutely conserved core structure in the CYP family by Yang et al. [24] and Huang et al. [25]. In our model, structural characteristics are quite different in the mutant than those in the WT, and these altered structural properties may be conducive to enzymatic function (Figure 4). Our results indicated that three hydrogen bonds between R390, E387, and N428 would be destroyed with arginine being replaced by histidine. Upon R390H mutation, there is an increase in the region of positive potential, indicating a significant rearrangement of the local charges around the 390th residue.

As residues 107 and 309 are very close to the functionally important regions ASC and HBR, it is expected that the mutation effects might extend way beyond the mutation sites and directly alter the molecular interactions between the CYP1B1 protein and the substrate. The mutation-induced changes are most likely to be key determinants in the altered function of the protein.

To verify this deduction, we conducted long-term MD simulations for the dynamics changes upon mutations. We found from the RMSD trajectories that the structures of the WT and mutants adopted different pathways of transition from the starting conformations to their final states, although the initial structures were almost identical except at the mutation sites. The RMSD data clearly indicate the destabilization effects of L107V and R390H mutations on the dynamics of CYP1B1 (Figure 5A). This conclusion was verified by the RMSF data, which indicated a subtle but significant increase in the flexibility of the molecule for the mutants (Figure 5B). Furthermore, the SASA of the ASC is smaller for both the mutants compared with WT, suggesting that some active sites were concealed by the mutations (Figure 5D). As the ASC of the enzymes must be fairly rigid to allow for specific interactions of functional groups and the substrate, the change in the SASA of the ASC of CYP1B1 might affect substrate binding as well as the enzyme activity and efficiency.

Generally, mutation predictions obtained using *in silico* tools should be interpreted with caution due to the extreme sensitivity of specific algorithm settings. It has been reported that more reliable results can be achieved when two or more tools are applied simultaneously [36,37]. In our prediction strategy, we used sequence conservation analysis, hydrogen bond network analysis, electrostatic potential analysis, and MD simulations to predict the impact caused by L107V and R390H mutations. The results supported each other and explained more clearly the effects of the mutations on the function of CYP1B1. However, though the two mutations have been reported in PCG patients of many populations, and the relationship between the *CYP1B1* gene variants and PCG pathogenesis was inferred in the present study, experiments using appropriate cell and animal models are required in the future to verify and explain how the mutations influence the protein’s function during eye development.

In conclusion, we found that L107V and R390H in CYP1B1 are two common mutations relating to PCG in Chinese populations. Both mutations may destroy the residue contacts, destabilize the local and/or whole protein, reduce the exposure of active sites, and finally affect the molecular interactions between CYP1B1 and the substrate. These results provide novel insights into genetic contribution to the etiology of PCG and can help to conduct functional experiments for the PCG-related mutations.

## Supporting information

**Figure S1.** Ramachandran plots of structural model of CYP1B1 mutants L107V and R390H. The most energetically allowed regions of Ramachandran space is colored blue and the allowed regions are colored green. This image was made with VMD.

**Table 1:** CYP1B1 gene mutation frequency in Chinese PCG patients

## Abbreviations

ASC: active site cavity
HBR: heme binding region
MD: molecular dynamics
PCG: primary congenital glaucoma
RMSD: root mean square deviation
RMSF: root mean square fluctuation
SASA: solvent-accessible surface area
WT: wild-type
