# Supplementary material for: Bioinformatics analysis of CYP1B1 mutation hotspots in Chinese primary congenital glaucoma patients
Source: Biosci Rep. 2018 Jul 6;38(4):BSR20180056. doi: 10.1042/BSR20180056 (PMC6435531; doi:10.1042/BSR20180056)
Supplement: Supplementary file 1 [file bsr20180056_Supp1.pdf]

Bioinformatics analysis of *CYP1B1* mutation hotspots in Chinese primary congenital glaucoma patients

Zhiying Ou[1,†], Guangjian Liu[1,†], Wenping Liu[2], Yehua Deng[3], Ling Zheng[3, 4], Shu Zhang[1], Guangqiang Feng[3,*]

[1]Institute of Pediatrics, Guangzhou Women and Children's Medical Center, Guangzhou Medical University, Guangzhou, Guangdong, China

[2]Department of Medical Devices, Guangdong Food and Drug Vocational College, Guangzhou, Guangdong, China

[3]Department of Ophthalmology, Guangzhou Women and Children's Medical Center, Guangzhou Medical University, Guangzhou, Guangdong, China

[4]Department of Ophthalmology, Jieyang People's Hospital, Jieyang Affiliated Hospital, Sun Yat-sen University, Jieyang, Guangdong, China

[*]Corresponding author: Guangqiang Feng, MD, Department of Ophthalmology, Guangzhou Women and Children's Medical Center, Guangzhou Medical University, 9 Jinsui Road, Tianhe District, Guangzhou 510623, China, Fax: 86 2038076072 Tel.: 86 2038076072 Email: 1012316516@qq.com

[†]These authors contributed equally to this work.

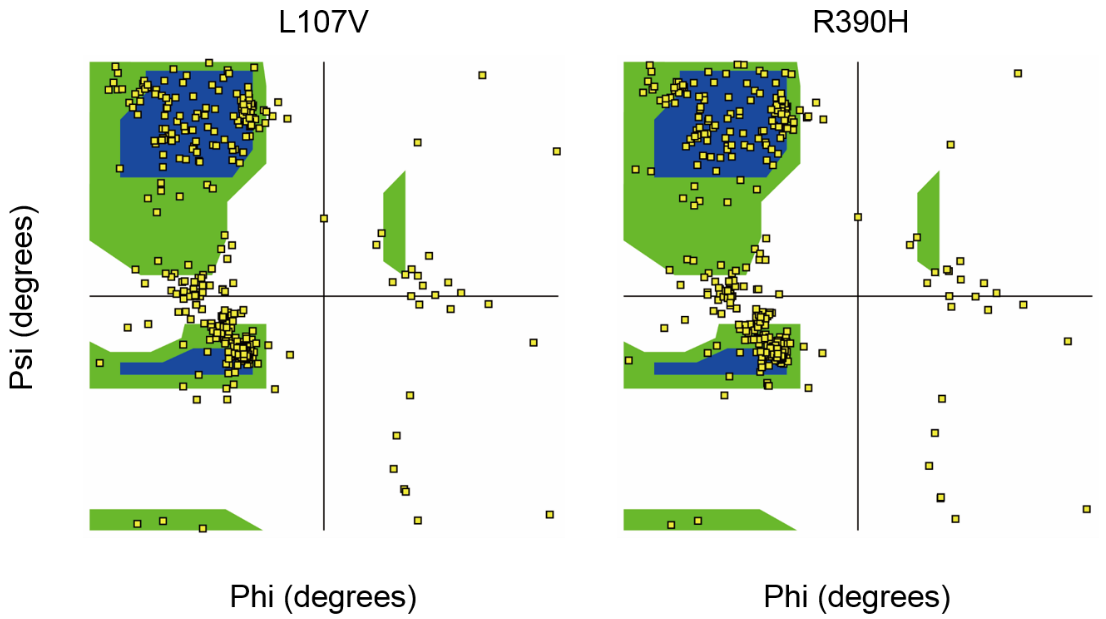

Figure S1. Ramachandran plots of structural models of CYP1B1 mutants L107V and R390H. The most energetically allowed regions of Ramachandran space is colored blue and the allowed regions are colored green. This image was made with VMD.

TABLE 1. CYP1B1 gene mutation frequency in Chinese PCG patients

Mutation Type	Nucleotide change	Amino acid	Positive cases/Total cases	First reported in China	Positive cases	Total cases	Mutation frequency (%)
	g.4124C>G	L107V	2/116[22];5/122[26]; 1/13[21]	No	8	251	3.19
	g.8006G>A	R390H	2/116[22];4/41[24]; 1/16[25];1/44[27]; 2/122[26]	No	10	324	3.09
	c.1007C>A	S336Y	1/41[24]	Yes	1	41	2.44
	c.1412T>G	I471S	1/41[24]	Yes	1	41	2.44
	g.7924G>T	V363F	1/44[27]	Yes	1	44	2.27
	g.7946C>T	P370L	1/44[27]	Yes	1	44	2.27
	g.8254G>A	E473K	1/44[27]	Yes	1	44	2.27
Missense mutation	g.4642C>G	H279Q	1/44[27]	Yes	1	44	2.27
	g.4677A>G	D291G	3/112[22]; 2/122[26]	Yes	5	234	2.14
	g.4763G>T	V320L	1/44[27]; 2/122[26]	No	3	166	1.81
	g.8242C>T	R469W	1/41[24];1/16[25]; 1/122[26]	No	3	179	1.68
	g.4338T>A	V178E	2/122[26]	Yes	2	122	1.64
	g.4493G>A	E230K	2/122[26]	Yes	2	122	1.64
	g.4449G>T	S215I	1/116[22]; 2/122[26]	No	3	238	1.26
	g.3985C>G	Z60M	1/116[22]	Yes	1	116	0.86
	g.4089T>C	V95A	1/116[22]	Yes	1	116	0.86
	g.4157C>T	P118S	1/116[22]	Yes	1	116	0.86
	g.4206T>C	F134S	1/116[22]	Yes	1	116	0.86
	g.4413A>G	N203S	1/116[22]	Yes	1	116	0.86
	g.4664G>A	A287S	1/116[22]	Yes	1	116	0.86
	g.4761A>G	N319S	1/116[22]	Yes	1	116	0.86
	g.7925T>A	V363D	1/116[22]	Yes	1	116	0.86
	g.4397G>A	V198I	1/116[22]	No	1	116	0.86
	g.8147C>T	P437L	1/116[22]	No	1	116	0.86
	g.7927G>A	V364M	1/116[22]; 1/122[26]	No	2	238	0.84
	g.8168G>A	R444Q	1/116[22]; 1/122[26]	No	2	238	0.84
	g.7940G>T	R368L	1/116[22]; 1/122[26]	Yes	2	238	0.84
	g.3836T>C	W11R	1/122[26]	Yes	1	122	0.82
	g.4151G>T	D116Y	1/122[26]	Yes	1	122	0.82
	g.4322G>A	E173K	1/122[26]	Yes	1	122	0.82
	g.4509T>C	V235A	1/122[26]	Yes	1	122	0.82
	g.8137T>C	W434R	1/122[26]	Yes	1	122	0.82
	g.8167C>T	R444stop	1/122[26]	Yes	1	122	0.82
Delete mutation	g.3972delC	A56GGHX→59stop	1/116[22]	Yes	1	116	0.86
	c.828delC	F276Ffsx1	1/41[24]	Yes	1	41	2.44
	c.726-747del22bp	D242Dfsx28	1/41[24]	Yes	1	41	2.44
	g.4022delTC	73ORF→221stop	1/122[26]	Yes	1	122	0.82
Insert mutation	g.4168_4169ins(GACCGGCCGG CCTTCGCC)	A121_S122ins(DRP AFA)	1/116[22]	Yes	1	116	0.86
Delete and Insert	g.8209_8213del(AGCAG)ins(TTG	S458_R459del,ins LLKK	1/116[22]	Yes	1	116	0.86

Mutation Type	Nucleotide change	Amino acid	Positive cases/Total cases	First reported in China	Positive cases	Total cases	Mutation frequency (%)
	TTGAAAAA)						
